# Conditional trust as a driver of public engagement in Korea’s national project of bio-big data

**DOI:** 10.3389/fgene.2025.1713598

**Published:** 2026-01-05

**Authors:** Jae Sun Kim, Seohyun Kim, Cheyeon Lee, Wonoh Jeong, Huiwon No, Sungsoo Lim, Yul Jung, Ilhak Lee, Kwangmo Yang

**Affiliations:** 1 College of Law, Dongguk University, Seoul, Republic of Korea; 2 Samsung Medical Center, Sungkyunkwan University School of Medicine, Seoul, Republic of Korea; 3 Department of Medicine, Sungkyunkwan University School of Medicine, Seoul, Republic of Korea; 4 Department of Computer Education, College of Education, Sungkyunkwan University, Seoul, Republic of Korea; 5 Department of Digital Health, Samsung Advanced Institute for Health Sciences and Technology (SAIHST), Sungkyunkwan University, Seoul, Republic of Korea; 6 Healthcare Research Division, Gallup Korea, Seoul, Republic of Korea; 7 Asian Institute for Bioethics and Health Law, Department of Medical Humanities and Social Sciences, College of Medicine, Yonsei University, Seoul, Republic of Korea; 8 Center of Health Promotion, Samsung Medical Center, Sungkyunkwan University School of Medicine, Seoul, Republic of Korea

**Keywords:** biobank, bio-big data, ELSI, NPBBD, public engagement, survey

## Abstract

**Introduction:**

Trust is built on the belief that promises will be honored. In biodata research, public trust is established when researchers conduct studies as promised and utilize the research outcomes in accordance with the stated objectives. Participants, particularly those contributing sensitive data, often demand a detailed explanation regarding how their data will be utilized and governed, which forms the foundation of trust. The National Project of Bio-Big Data (NPBBD) was conceived upon this premise. Within this framework, participants recognize the necessity of biobanks, the state ensures trustworthy governance through institutional safeguards, and researchers uphold these commitments—thereby sustaining a cycle of trust. Although prior studies have examined public attitudes toward biobanks and general willingness to participate, less is known about their perspectives on consent models, governance structures, benefit-sharing mechanisms, and motivations for engagement. Persistently low participation rates highlight the need for systematic analysis of underlying barriers and strategies to strengthen public involvement. Considering the NPBBD’s goal of building a nationwide cohort of one million individuals, this study seeks to identify the motivating factors that can foster improved public participation. By assessing public awareness and participation drivers, we aim to delineate the conditions of trust from the participants’ perspectives and offer guidance for data-driven policies grounded in medical, ethical, legal, and social legitimacy.

**Methods:**

Between August 22–30, 2024, a web- and mobile-based survey was conducted among 1,027 adults aged 19–64 years, residing across 17 provinces and metropolitan cities in Korea, selected via proportional allocation. The questionnaire, developed with reference to prior studies including ethical, legal, and social implications, comprised 19 items across six domains, and 16 sociodemographic questions.

**Results:**

Overall, 15.1% of the respondents were aware of the NPBBD, and 35.1% had heard of it, as compared to 49.1% who recognized the term “biobank.” Willingness to participate varied by incentive: 60.9% expressed willingness to participate when personal benefits were offered, 29.9% were motivated by public interest, and 9.3% declined participation altogether. Among the non-participants, 18.9% agreed to participate when presented with a rare disease scenario. Anticipated benefits included early detection and prevention (mean score: 78.3), treatment for rare or incurable diseases (76.5), and advancement of research (75.4). Key concerns centered on data breach (77.0), third-party misuse (76.1), and unconsented data use (75.6). Participation drivers included contribution to science (74.6), support for future generations (72.5), and personal benefits (68.1), with access to professional consultation (78.6) emerging as the most influential factor. The most desired information was genetic risk prediction (62.8%). Respondents favored receiving updates on sanctions for misuse (82.0%), supervisors assigned to monitor data use (80.3%), and ongoing research use (75.0%), through text messages (41.0%) and email (36.1%). Regarding additional data, 39.6% were willing to share information about their health, lifestyle, and habits; 38.0% preferred to share health information only; and 13.2% were ready to give biospecimens only. While the majority of participants favored reinvesting profits into drug and treatment development, others preferred receiving benefits through partial coverage of treatment costs (64.1%). In terms of governance, most respondents preferred a shared model involving government, public, and private institutions (44.7%). Consent preferences leaned toward dynamic (57.8%) over broad consent (35.2%).

**Discussion:**

Public perception of biodata collection reflected a mix of anticipated personal and public benefits, alongside concerns regarding data disclosure. Willingness to participate was influenced more by personal benefits (60.9%) than by public interest (29.9%). While personal incentives such as treatment cost coverage were valued, participation was also driven by a desire to contribute to research and support drug development, reflecting a blend of self-interest and altruistic motivation. Concerns centered on data leakage, with dynamic consent emerging as a key condition of trust, alongside public governance and the right to information. To secure public trust and increase participation in the NPBBD, tailored strategies must be used to balance personal and public benefits with transparent governance, information provision, and consent procedures.

## 1 Introduction

Trust is built on the belief that promises will be honored. In biodata research, public trust is established when researchers conduct the study as promised and utilize the research outcomes in accordance with the stated objectives. Participants, particularly those contributing sensitive data, often demand detailed conditions that form the foundation of trust. The National Project of Bio-Big Data (NPBBD) was established on this premise. Within this framework, participants acknowledge the necessity of biobanks, the state ensures governance through institutional safeguards, and researchers maintain these commitments—sustaining a cycle of trust.

With recent advancements of the bio-health industry, the medical paradigm is transitioning from traditional empirical and intuition-based approaches toward predictive, preventive, personalized, and participatory care (Lee and Kim, 2024). This transition necessitates the establishment of a precision medicine-based healthcare system that integrates large-scale bio-big data with clinical, genomic, and lifestyle information. A biobank serves as a crucial infrastructure for storing and managing physical biological specimens (e.g., blood, tissue, DNA) along with related clinical information. Meanwhile, bio-big data refers to integrating and analyzing digital datasets derived from these specimens, including genomic, clinical, and lifestyle data. In essence, a biobank is a repository of biological resources, whereas bio-big data is an infrastructure that transforms those resources into knowledge. Korea’s National Bio-Big Data (NPBBD) initiative builds on biobank resources to advance data-driven research, focusing on fostering the ethical use of data and public trust rather than the physical preservation of samples. Currently, Korea faces a critical shortage of integrated bio-big data for both medical and industrial applications. Researchers are often compelled to independently build or collect data for their specific objectives. The lack of standardized institutional procedures, utilization frameworks, and cost considerations further hinders the efficiency and scalability of data analysis. Moreover, data generated at hospital or institutional levels often lack standardization and interoperability, underscoring the urgent need for a national strategic data infrastructure (Ryu et al., 2023).

In response to these challenges, the Ministry of Health and Welfare, together with the Ministry of Science and ICT, the Ministry of Trade, Industry and Energy, and the Korea Disease Control and Prevention Agency, jointly launched the NPBBD. This flagship initiative aims to collect bio-big data from one million individuals (400,000 patients and 600,000 healthy individuals) over a 9-year period (2024–2032), across three strategic phases. The overarching goal of this endeavor is to accelerate innovation in precision medicine and the bio-industry. Phase 1 (2024–2026) involves the establishment of a clinical and genomic database; in Phase 2 (2027–2029), the database will be extended to include disease-specific omics data; finally, in Phase 3 (2030–2032), healthcare professionals will be provided with access to fully a integrated bio-big database (Kim, 2023). Following a successful preliminary feasibility review in 2023, the project aims to secure data from approximately 770,000 individuals by 2028. This initiative adopts a systematic approach to streamline the often complex informed consent process and foster voluntary public participation (Jung et al., 2023).

Against this backdrop, the current study qualitatively examines public perceptions and attitudes toward the NPBBD, drawing on findings from a 2024 national perception survey. Given its unprecedented scale and pace of execution, the NPBBD is regarded as a touchstone for the Korean Ethical, Legal, and Social Implications (ELSI) policy. The key factors assessed in this study include voluntary participation, personal information protection, and trust in benefit-sharing mechanisms. Korea’s genomics-based healthcare policy is shifting from a regulatory-centric legal framework (e.g., the Bioethics and Biosafety Act) to a data-driven governance model (Park et al., 2024). Consequently, ELSI concerns such as consent systems, secondary data use, and prevention of genetic discrimination are being redefined in light of public acceptability. Furthermore, recent legislative developments, such as the Advanced Regenerative Medicine Act, are reshaping research participation and data-sharing mechanisms, underscoring the need for policy design aligned with these institutional changes (Republic of Korea, 2020).

In Korea, consent has often been treated as a procedural formality, with limited explanation provided regarding data use. This has led to low public trust in sharing sensitive information. The NPBBD was established to address this concern, aiming not merely to secure data, but to promote voluntary participation grounded in public trust. Accordingly, beyond measuring acceptability, this study seeks to identify the practical conditions and public expectations to be fulfilled by a national bio-big data initiative to attain ethical, legal, and social legitimacy and long-term sustainability. It aims to identify factors that encourage or hinder participation, while also exploring how trust in data security can strengthen public engagement. Ultimately, the study aims to propose ethical and social foundations for future Korean ELSI policies, enabling genomics-based precision medicine to balance public good with the protection of individual rights.

The successful implementation of large-scale, government-led biodata initiatives hinges on participant trust. Participation in NPBBD will require the disclosure of sensitive information, including personal information, genomic data, and medical history, from patients with chronic or rare conditions as well. Therefore, it is important for participants to provide informed consent after gaining a clear understanding of the initiative, its benefits, risks, and potential outcomes. Trust in data use, privacy protection, and research purpose legitimacy are critical for the success of NPBBD. Consequently, conducting surveys to evaluate public willingness to participate in the NPBBD is essential for policy development and project implementation.

The objectives of this study are: 1) To analyze public awareness and understanding of the NPBBD, including their perception of its goals and data utilization policies; 2) To identify expectations and concerns affecting participation; 3) To examine levels of trust, willingness to participate, and attitudes toward benefit-sharing mechanism. Based on these findings, the study will propose recommendations to enhance public participation through institutional improvements in information provision, governance, benefit allocation, consent procedures, and data management.

## 2 Materials and methods

### 2.1 Survey methods

The survey was administered online via web and mobile platforms. Participants were recruited from a professional research company and comprised men and women aged 19–64 years residing across 17 cities and provinces in Korea. The sampling employed proportional allocation by gender, age (in 5-year intervals), and region to ensure representativeness. The exclusion criteria were applied prior to and following data collection. Participants were excluded prior to the survey if they had participated in studies on a similar topic within the previous 6 months. Post-hoc exclusions included respondents whose data indicated questionable reliability or sincerity, as determined by survey completion time, response consistency, and the repeated selection of identical scale options. After applying these criteria, a total of 1,027 valid responses were retained over a 9-day period (August 22–30, 2024). The margin of error was ±3.1% at the 95% confidence level.

### 2.2 Questionnaire development

The questionnaire was developed with reference to studies by Kim et al. (2023) and Yang et al. (2023), while incorporating insights from the ELSI of large-scale biobanking projects and recent debates on participant engagement. Prior research has highlighted that ELSI in Korea is largely institutionalized, with limited citizen participation (Lee, 2022). To address this gap, it is essential to capture the perceptions, concerns, and expectations of those directly involved, particularly with regard to consent. Unlike earlier studies that primarily evaluated willingness to participate, this study aimed to develop proactive engagement strategies by comprehensively assessing attitudes, expectations, concerns, motivations, compensation preferences, and information-sharing behaviors.

The questionnaire included 19 items across six thematic domains, presented in single-choice, multiple-choice, and five-point scale formats, and 16 sociodemographic questions (e.g., gender, residence, age, family size, education, marital status, occupation, subjective health and living standards, income, etc.). The six domains were: 1) awareness of the NPBBD; 2) willingness to participate; 3) expectations and concerns regarding participation; 4) factors influencing decision to participate; 5) benefits and information desired; and 6) governance and operation of the NPBBD.

### 2.3 Statistical analysis

Analyses were performed using SPSS version 26.0 (IBM Corp., Armonk, NY, USA). Incomplete responses were excluded from the analysis. Chi-square tests were applied, with significance set at p < 0.05.

### 2.4 Ethical considerations

This study was reviewed and approved by the Institutional Review Board of Samsung Medical Center (Approval No. 2025-09-112). All participants were informed in advance of the study’s objectives, the voluntary nature of their participation, and the confidentiality measures. They were assured that their responses would remain anonymous and be used solely for research purposes. The collected data were stored on secure servers with restricted access, in compliance with relevant ethical guidelines and the Personal Information Protection Act of Korea.

## 3 Results

### 3.1 Respondent characteristics and personalities

#### 3.1.1 Respondent characteristics

The sociodemographic characteristics of the respondents, including sex, age, and residential area, are presented in Table 1, along with household-related factors such as single-person household, number of household members, and marital status. Educational and employment factors were also considered, including experience of graduate school and employment status. In addition, self-perceived living standard, monthly household income (KRW), and private insurance status were examined. Finally, the respondents were asked about health-related experiences, including rare disease and cancer diagnoses (self or family).

**TABLE 1:** Sociodemographic characteristics of respondents (n = 1,027).

Total		N	%	Total		N	%	Total		N	%
		(1,027)	100.0			(1,027)	100.0			(1,027)	100.0
Sex	Male Female	(521) (506)	50.7 49.3	Single-person household	Yes No	(133) (894)	13.0 87.0	Private insurance status	Have medical insurance Do not have medical insurance/Do not remember	(836) (120) (71)	81.4 11.7 6.9
Age	20–29 30–39 40–49 50–59 60 and above	(193) (199) (248) (259) (128)	18.8 19.4 24.1 25.2 12.5	Members in household	1 2 3 4 5 or more	(133) (187) (353) (301) (53)	13.0 18.2 34.4 29.3 5.2	Rare disease diagnosis (self or family)	Have experienced Have not experienced	(135) (892)	13.1 86.9
Residential areas	Seoul Busan Daegu Incheon Gwangju Daejeon Ulsan Sejong Gyeonggi Gangwon Chungbuk Chungnam Jeonbuk Jeonnam Gyeongbuk Gyeongnam Jeju	(197) (67) (47) (62) (31) (31) (22) (4) (283) (25) (31) (40) (31) (30) (46) (62) (18)	19.2 6.5 4.6 6.0 3.0 3.0 2.1 0.4 27.6 2.4 3.0 3.9 3.0 2.9 4.5 6.0 1.8	Marital status	Single Married (including common-law) Separated/Divorced/Widowed	(413) (573) (41)	40.2 55.8 4.0	Rare disease diagnosis (self or family) 2	Never diagnosed Declared cured after treatment Currently undergoing treatment Untreated despite diagnosis	(892) (42) (52) (41)	86.9 4.1 5.1 4.0
				Experience of graduate school	Yes No	(883) (144)	86.0 14.0				
				Employment status	Employed Unemployed	(751) (276)	73.1 26.9	Cancer disease diagnosis (self or family)	Have experienced Have not experienced	(291) (736)	28.3 71.7
				Self-perceived living standard	High Medium Low	(159) (525) (343)	15.5 51.1 33.4				
				Self-perceived health	Poor Fair Good	(161) (562) (304)	15.7 54.7 29.6	Cancer disease diagnosis (self or family) 2	Never diagnosed Declared cured after treatment Currently undergoing treatment Untreated despite diagnosis	(736) (158) (96) (37)	71.7 15.4 9.3 3.6
				Monthly household income (KRW)	<2 million 2–4 million 4–6 million 6–8 million ≥8 million	(86) (268) (264) (219) (190)	8.4 26.1 25.7 21.3 18.5				

##### 3.1.1.1 Awareness of NPBBD and biobank

Participant responses to the question about their awareness of NPBBD included: “I know it,” “I have heard of it,” and “I do not know it/I have not heard of it.” Responses to a parallel question about awareness of biobanks were: “I have heard of it” and “I do not know it/I have not heard of it.”

**FIGURE 1:**
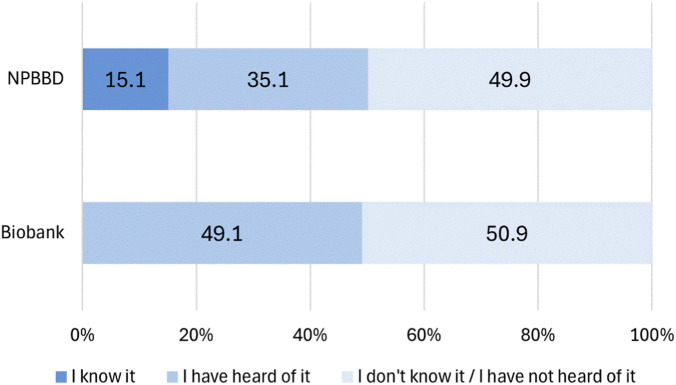
Awareness of the NPBBD and biobanks (*n* = 1,027). The graph shows public awareness levels of the NPBBD and biobanks. The light blue bar represents respondents who “know about it,” the medium blue bar represents those who “have heard of it,” and the gray bar represents those who “do not know about it/have not heard of it.”

As shown in Figure 1, 15.1% of the respondents reported being aware of the NPBBD. The percentages of respondents who had heard of NPBBD and biobanks were 35.1% and 49.1%, respectively, while those who had not heard of them accounted for 49.9% and 50.9%, respectively. Compared to the results of a similar survey conducted 3 years earlier, awareness of biobanks had increased, whereas awareness of the NPBBD had decreased (Yang et al., 2023). Nevertheless, the percentage of respondents who “knew” about the NPBBD increased by 3.9% compared with the results of the earlier survey (Yang et al., 2023).

##### 3.1.1.2 Rate of participation in the human biospecimen collection pilot project

Respondents were asked whether they had participated in the Ministry of Health and Welfare’s 2020–2022 pilot project for human biospecimen collection (blood, urine, and tissue). The response options—“Yes,” “No,” and “Unsure”—were selected by 11.3%, 78.3%, and 10.4% of the participants, respectively (Table 2).

**TABLE 2:** Participation in the Human Biospecimen Collection Pilot Project (*n* = 1,027), categorized by awareness of the NPBBD and biobank and by perceptions toward participation.

Variable	Categories	Total	Yes	No	Unsure	p-value
		N	N (%)	N (%)	N (%)	
All	​	1027	116 (11.3)	804 (78.3)	107 (10.4)	​
Awareness of the NPBBD	I Know it	155	77 (49.7)	72 (46.5)	6 (3.9)	<0.001
	I Have heard of it	360	39 (10.8)	286 (79.4)	35 (9.7)	
	I do not know it/I have not heard of it	512	0 (0)	446 (87.1)	66 (12.9)	
Awareness of the biobank	I Have heard of it	504	111 (22.0)	349 (69.2)	44 (8.7)	<0.001
	I do not know it/I have not heard of it	523	5 (1.0)	455 (87.0)	63 (12.0)	
Perceptions towards participation in the NPBBD	Expectations outweigh concerns	159	14 (8.8)	126 (79.2)	19 (11.9)	0.004
	Concerns and expectations are similar	461	40 (8.7)	363 (78.7)	58 (12.6)	
	Concerns outweigh expectations	407	62 (15.2)	315 (77.4)	30 (7.4)	

Overall, awareness of NPBBD and biobanks, as well as expectations and concerns about NPBBD, were observed to have a significant effect (*p* < 0.05) on participation behavior in the pilot project. Among those who “knew” about the NPBBD, 49.7% reported having participated in the pilot project, which is substantially higher than the overall rate (11.3%). Furthermore, 15.2% of those who reported that their expectations about NPBBD outweighed their concerns participated, compared to only 8.8% participation from those who reported that their concerns outweighed their expectations. This suggests that positive expectations are linked to behavioral engagement.

### 3.2 Factors affecting willingness to participate

#### 3.2.1 Willingness to participate in the NPBBD

**FIGURE 2:**
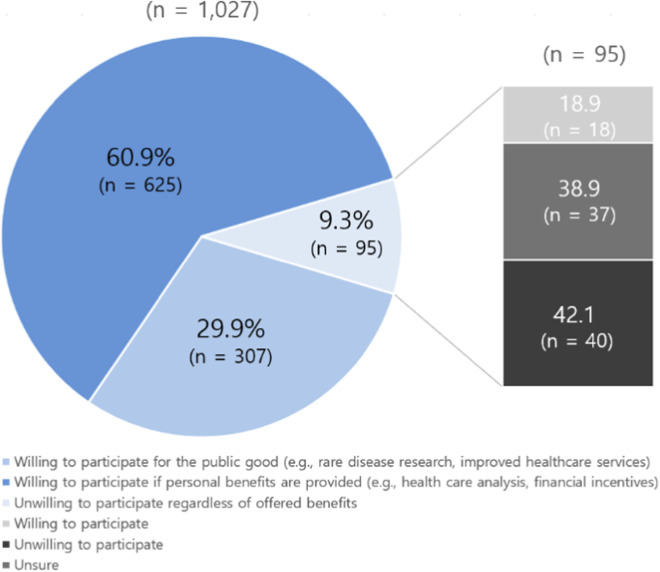
Respondents’ willingness to participate in the NPBBD (left, *n* = 1,027); reassessed willingness of respondents initially unwilling to participate after being presented with the hypothetical scenario of a family member being affected by a rare disease (right, *n* = 95).

When asked about willingness to participate in the NPBBD, 60.9% (n = 625) of the participants expressed willingness if personal benefits such as healthcare analysis and financial support were provided; 29.9% (n = 307) indicated that they would be willing to participate for public good (e.g., rare disease research, improvement of healthcare services); and 9.3% (n = 95) responded that they were not willing to participate regardless of the benefits offered (Figure 2). After the participants were informed that the data accumulated in the biobank would be used for research, development of precision medicine, and drug development for rare and severe diseases, those who had initially expressed unwillingness were asked whether they would consider participating if a family member had a rare disease. In response to this question, 18.9% (n = 18) changed their answer to willing, 42.1% (n = 40) remained unwilling, and 38.9% (n = 37) expressed uncertainty.

Categorization of willingness to participate as per demographic characteristics and awareness is provided in Supplementary Table S3. Among men, 34.2% reported willingness to participate for the public good, which was higher than the overall average. Among women, both the proportion willing to participate if personal benefits were offered and the proportion unwilling to participate exceeded the overall average. Participation for public good increased with age, peaking at 42.2% among respondents aged 60 or above. Awareness also mattered: those familiar with the NPBBD were more likely to participate for public benefit, than those with less awareness. Similarly, those who had participated in the human biospecimen collection pilot project demonstrated greater inclination toward participating for public benefit than those who had not. Awareness of biobanks was also significant: those who had heard of biobanks were more likely to participate, while those who had not were markedly more likely to refuse (*p* < 0.001).

Respondents with greater concerns than expectations exhibited reduced willingness to participate for public good. However, willingness to participate if offered personal benefits was highest among participants whose expectations and concerns were balanced. Notably, the difference in willingness between respondents with greater expectations and those with greater concerns was only 4.6%, suggesting that incentives could effectively increase participation.

Insurance and socioeconomic status were also noted to influence willingness. Respondents with private health insurance expressed higher intention to participate for the public good (32.1%) compared to those without insurance (24.2%). Unemployed individuals reported significantly higher rates of non-participation compared to employed respondents. Perceived socioeconomic status also shaped willingness: those with higher self-rated living standards and higher household income were more likely to participate for public benefit, with the highest level of willingness (38.9%) observed among participants whole household earning was more than eight million KRW per month.

#### 3.2.2 Expectations and concerns

**FIGURE 3:**
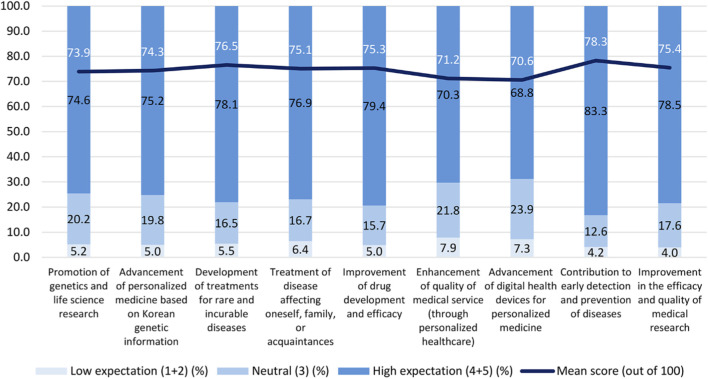
Expected regarding the benefits of participating in the NPBBD (*n* = 1,027). Rated on a 5-point Likert-type scale with mean scores (0–100). The assessment covered various areas, including the promotion of genetics and life science research, the advancement of personalized medicine based on Korean genetic information, the development of treatments for rare and incurable diseases, the treatment of diseases affecting oneself and family members, improved drug development and efficacy, the enhancement of medical service quality through personalized healthcare, and the advancement of digital health devices.

The respondents were asked to rate their level of expected benefits of participation on a five-point scale (1 = “Not at all expected” to 5 = “Highly expected”). Figure 3 presents participants’ expectations as mean scores (white labels, 0–100 scale) and response distributions (black labels, low concern = scores 1–2, neutral = score 3, high concern = scores 4–5). Overall, expectations were positive. As shown in Figure 3, the highest expectations were early detection and prevention of disease (78.3%), development of treatments for rare and previously incurable diseases (76.5%), and improvement in the efficacy and quality of medical research (75.4%). Across all outcome categories, the proportion of respondents with high expectations (scores of 4–5) was the largest (68.8%–83.3%), with the highest level of expectations observed for early detection and prevention of diseases (83.3%), followed by improvement in the efficacy and quality of medical research (78.5%) and development of treatments for rare and previously incurable diseases (78.1%). Neutral responses (scores of 3) were relatively more frequent for outcomes related to systemic or technology-driven aspects, such as advancements in digital health devices (23.9%) and enhancement of medical service quality (21.8%), indicating greater uncertainty. Conversely, low expectations (scores of 1–2) were minimal across all categories (4.0%–7.9%), with the lowest levels reported for improvement to medical research (4.0%) and disease prevention (4.2%). Collectively, these findings suggest that the participants placed particularly valued tangible, patient-centered outcomes such as prevention, treatment, and research advancement while showing comparatively more cautious attitudes toward systemic improvements and the development and integration of digital health technologies.

**FIGURE 4:**
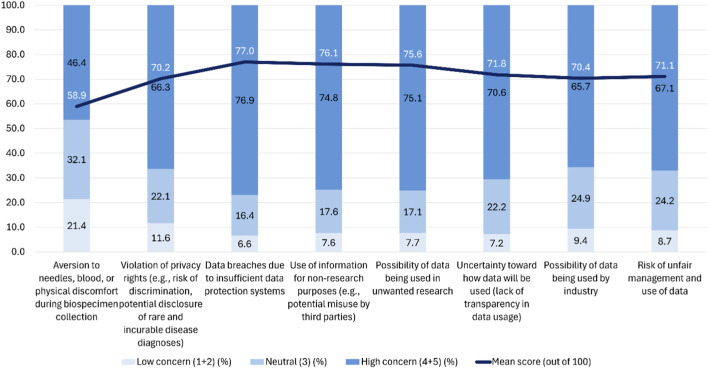
Concerns about participation in the NPBBD (*n* = 1,027). Rated on a 5-point Likert-type scale with mean scores (0–100). The assessment covered various issues, including aversion to needles, blood, or physical discomfort during biospecimen collection, the violation of privacy rights (e.g., risks of discrimination, disclosure of rare or incurable disease diagnoses), data breaches due to insufficient protection systems, the use of information for non-research purposes or potential misuse by third parties, the possibility of one’s data being used in unwanted research, uncertainty and lack of transparency regarding data usage, the potential use of data by the industrial sector, and risks of the unfair management or exploitation of data.

The participants were asked to rate their concerns regarding participation on a similar scale. As shown in Figure 4, based on mean scores, the highest concerns were related to data governance, including “data breaches due to insufficient data protection systems (77.0%), followed by “use of information for non-research purposes (e.g., potential misuse by third parties)” (76.1%) and “possibility of data being used in unwanted research” (75.6%) and “violation of privacy rights (e.g., risk of discrimination, potential disclosure of rare and incurable disease diagnoses)” (70.2%). In contrast, only 58.9% expressed considerable concern over physical discomfort, such as aversion to needles, during biospecimen collection. Neutral responses (score 3) were most frequent regarding aversion to needles (32.1%), industrial use of data (24.9%), risks of unfair data management (24.2%), and uncertainty about transparency in data usage (22.2%), suggesting ambivalence rather than outright rejection. Low concern (scores 1–2) was generally minimal for governance-related items (e.g., 11.6% for privacy violations) but more pronounced for biospecimen collection discomfort (21.4%). Taken together, these findings indicate that the respondents’ primary anxieties are concentrated on data security, transparency, and potential misuse, whereas procedural discomfort associated with biospecimen collection is comparatively less salient. This observation underscores the need for robust protection systems and transparent communication regarding data use to build and sustain public trust.

### 3.3 Expected benefits of participation

#### 3.3.1 General motivation for decision to participate

**FIGURE 5:**
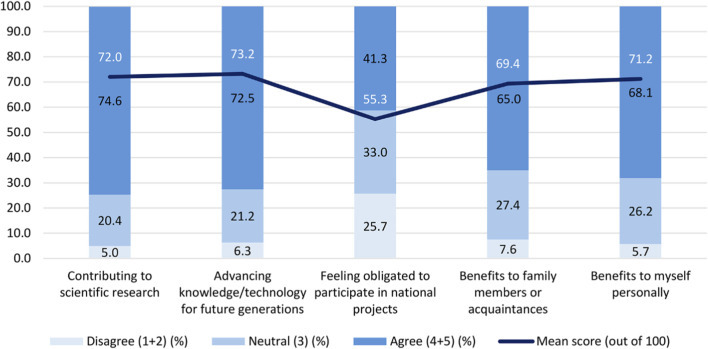
Motives for participating in the NPBBD (*n* = 1,027). Rated on a 5-point Likert-type scale with mean scores (0–100). The assessment covered several motivating factors, including contributing to scientific research, advancing knowledge and technology for future generations, fulfilling a sense of duty to participate in a national project, providing benefits to family members or acquaintances, and obtaining personal benefits.

As shown in Figure 5, the strongest motivations for participating in the NPBBD were “advancing knowledge/technology for future generations” (73.2%), “contributing to scientific research (72.0%), “benefits to myself personally” (71.2%), and “benefits to family members or acquaintances” (69.4%). In contrast, “feeling obligated to participate in national projects” scored much lower (55.3%). The stacked bar distribution further highlights that disagreement (scores 1–2) was minimal across most motivational domains (5.0%–7.6%), except for national obligation, where over one-quarter (25.7%) of the participants expressed dissent. These results suggest that participation is primarily driven by intrinsic and relational motivations, such as commitment to scientific progress and social value, rather than a sense of national obligation.

#### 3.3.2 Helpful benefits influencing decision-making

**FIGURE 6:**
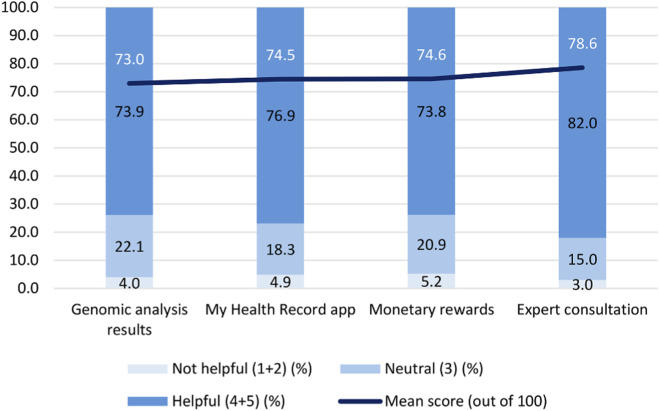
Influence of perceived benefits on participation decisions. Rated on a 5-point Likert-type scale with mean scores (0–100). The assessment covered genomic results, health apps, monetary rewards, and expert consultation, with responses categorized as “Not helpful” (1–2), “Neutral” (3), and “Helpful” (4–5).

When asked about factors that could influence the participants’ decision to participate, the highest scoring option was “the opportunity to consult with a specialist upon disease detection” (78.6%) (Figure 6). This option exceeded monetary incentives such as event coupons (74.6%), medical record management via the My Health Record app (74.5%), and genomic analysis results (73.0%). Specialist feedback was also the only item rated as “helpful” by more than 80% of respondents, highlighting that long-term, interactive healthcare engagement is a critical motivator.

#### 3.3.3 Desired information and benefits for participation

**FIGURE 7:**
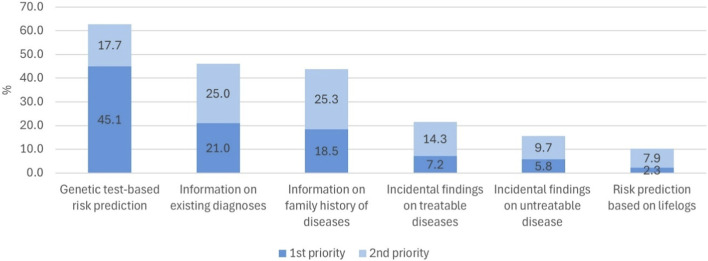
Priorities regarding health-related information and benefits for participating in NPBBD (*n* = 1,027). This bar graph illustrates the percentages of respondents who selected each result type as their first and second preferences for the NPBBD project.

The participants were asked to select up to two types of health-related information they would most like to receive if they joined the NPBBD. As shown in Figure 7, the strongest preference was for genetic disease risk prediction (1st: 45.1%, 1st + 2nd: 62.8%), reflecting interest in forecasting future health conditions. This was followed by information on existing diseases or conditions (1st: 21.0%, 1st + 2nd: 46.1%), and family history of disease (1st: 18.5%, 1st + 2nd: 43.8%). Lower preferences included incidental findings for treatable diseases (1st: 7.2%, 1st + 2nd: 21.5%), incidental findings for untreatable or hard-to-treat diseases (1st: 5.8%, 1st + 2nd: 15.6%), and disease risk prediction based on lifelog data such as heart rate or step count (1st: 3.2%, 1st + 2nd: 10.2%).

#### 3.3.4 Categories of information desired as data subjects

**FIGURE 8:**
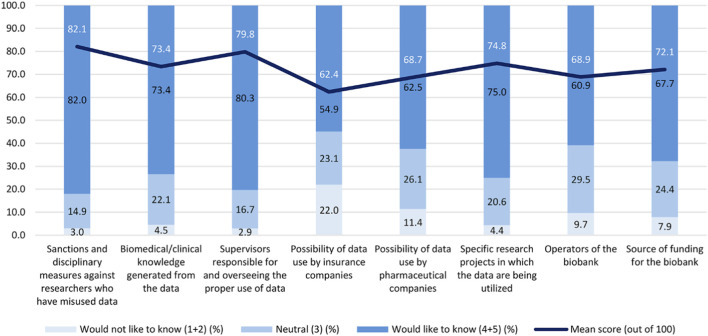
Interest in data transparency and governance information from the NPBBD (*n* = 1,027). Rated on a 5-point Likert-type scale with mean scores (0–100). The assessment covered sanctions and disciplinary measures against researchers who have misused data, biomedical and clinical knowledge generated from the data, supervisors responsible for proper data use, the possibility of data use by insurance companies, the possibility of data use by pharmaceutical companies, the information being utilized in ongoing research, biobank operators, and the source of funding for the biobank.

When asked about information desired as NPBBD participants, the strongest demand was for disclosure of “sanctions and disciplinary measures against researchers who have misused data” (82.1%), followed by information on “supervisors responsible for and overseeing the proper use of data” (79.8%), and “specific research projects in which the data are being utilized” (74.8%). Figure 8 illustrates these preferences.

A statistically significant difference was observed between demand for information on sanctions and demand for research-use details (*p* < 0.001, *p* = 0.00066), indicating stronger public interest in accountability and punishment systems than in transparency of research data utilization.

### 3.4 Preferences as data subjects

#### 3.4.1 Preferences as data subjects

**FIGURE 9:**
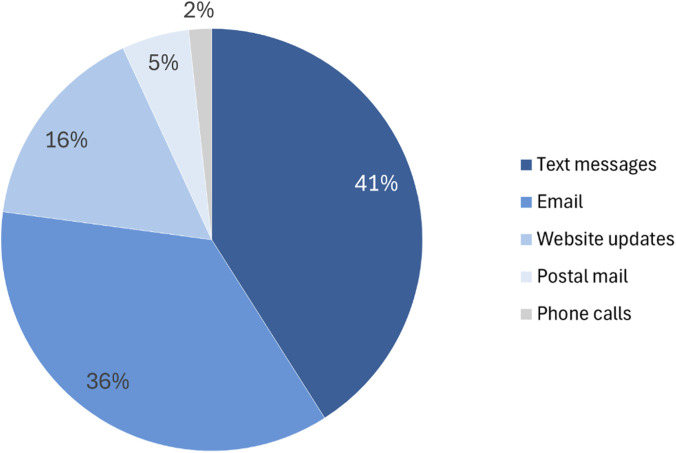
Preferred methods for receiving NPBBD-related information (*n* = 1,027). The assessment covered various methods, including text messages, email, website updates, postal mail, and phone calls.

As shown in Figure 9, respondents expressed clear preferences for digital communication in their answers to the survey question on the preferred methods of receiving NPBBD-related information. Specifically, 41.0% chose text messages and 36.1% chose email, together accounting for 77.1% of all responses. Less favored channels included website updates (16.0%), postal mail (5.2%), and phone calls (1.8%). These results indicate that participants viewed text messages and email as the most effective channels for receiving personalized health information, consent-related notifications, and information on data usage.

**FIGURE 10:**
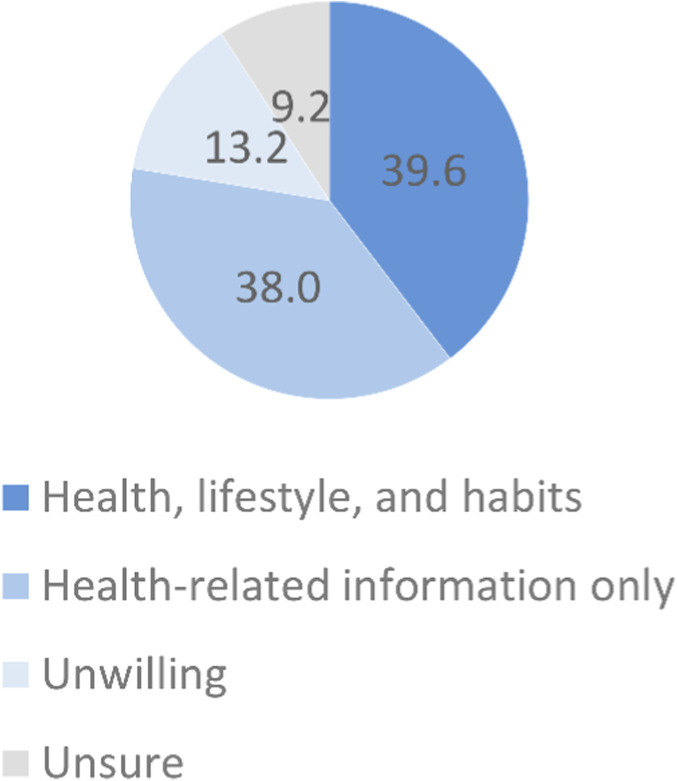
Willingness to share additional data beyond biospecimens with the NPBBD (*n* = 1,027). The pie chart shows participants’ responses regarding their willingness to share additional personal information beyond biospecimens.

When asked whether they would be willing to provide health, lifestyle, and habit data in addition to biospecimens, 39.6% responded affirmatively (Figure 10), reflecting recognition of the project’s public value and the need for integrated data. In contrast, 38.0% were willing to provide health information only, excluding lifestyle and habit data, indicating ongoing privacy concerns. A further 13.2% were unwilling to provide any additional information beyond biospecimens, preferring minimal participation.

#### 3.4.2 Preferred mode of provision of benefits

**FIGURE 11:**
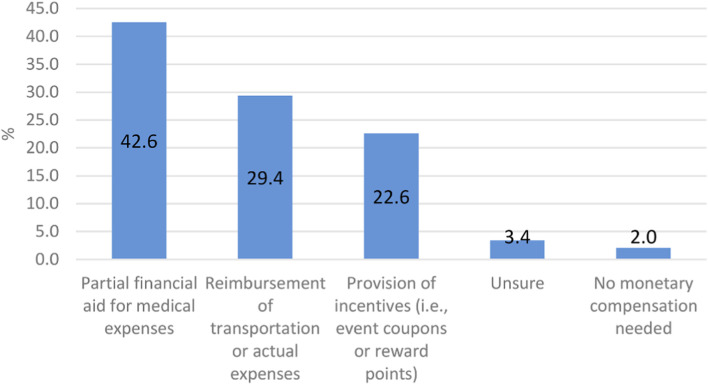
Preferences for compensation for participation in the NPBBD (*n* = 1,027). This bar graph shows the most appropriate forms of monetary compensation as selected by respondents.

Respondents were asked: “If monetary compensation were to be considered in relation to your participation in the NPBBD, which of the following options do you consider most appropriate?” As shown in Figure 11, the most frequent choice was partial financial aid for medical expenses (42.6%), followed by reimbursement of transportation or actual expenses (29.4%), and incentives such as event coupons or reward points (22.6%). A smaller proportion answered “unsure” (3.4%), while 2.0% indicated that they were not looking for any monetary compensation.

#### 3.4.3 Opinion on utilization of return on research

**FIGURE 12:**
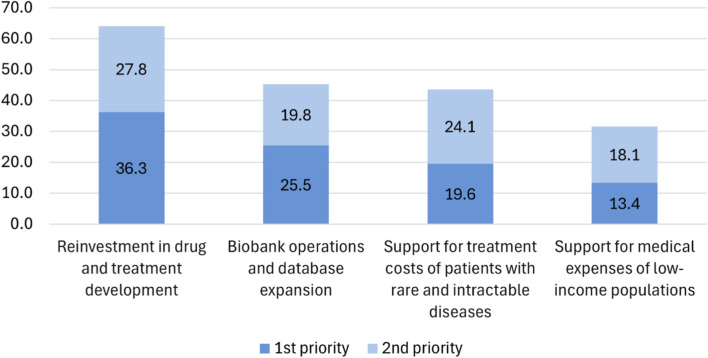
Priorities for the utilization of research returns for the NPBBD (*n* = 1,027). The bar graph indicates that participants most strongly preferred reinvestment in drug and treatment development, followed by biobank operations and database expansion, financial support for the treatment of patients with rare and intractable diseases, and support for the medical expenses of low-income populations.

When asked how profits generated by the biobank should be used (Figure 12), the top preference was “reinvestment in drug and treatment development” (1st: 36.3%, 1st + 2nd: 64.1%). This was followed by “biobank operations and database expansion” (1st: 25.5%, 1st + 2nd: 43.6%), “support for treatment costs of patients with rare and intractable diseases” (1st: 19.6%, 1st + 2nd: 45.3%), and “support for medical expenses of low-income populations” (1st: 13.4%, 1st + 2nd: 31.5%).

#### 3.4.4 Information governance

##### 3.4.4.1 Operating entity

**FIGURE 13:**
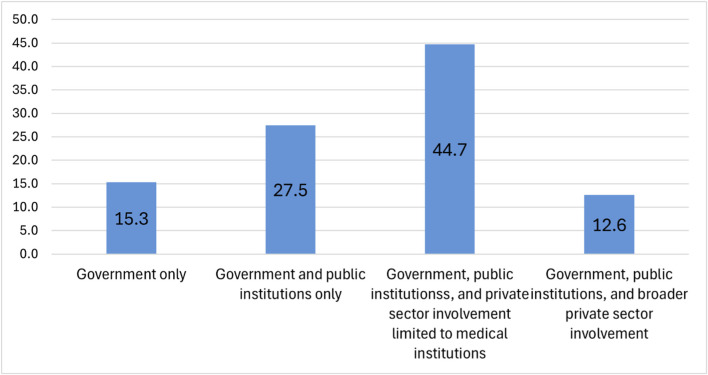
Preferred operating entities for data governance in the NPBBD (*n* = 1,027). The bar graph shows that most respondents preferred governance to be undertaken by government and public institutions, and private sector involvement to be limited to medical institutions. This was followed by preferring government and public institutions only, preferring government only, and preferring government, public institutions, and broader private sector involvement.

Regarding governance, 44.7% of the respondents expressed a preference for government and public institutions, with limited involvement from private sector restricted to medical institutions (Figure 13). Other responses were: government and public institutions only (27.5%), government only (15.3%), and government, public institutions, and broader private sector involvement including medical institutions and private companies (12.6%). This indicates a clear preference for public-sector leadership, with limited and cautious inclusion of private sector.

##### 3.4.4.2 Consent model

**FIGURE 14:**
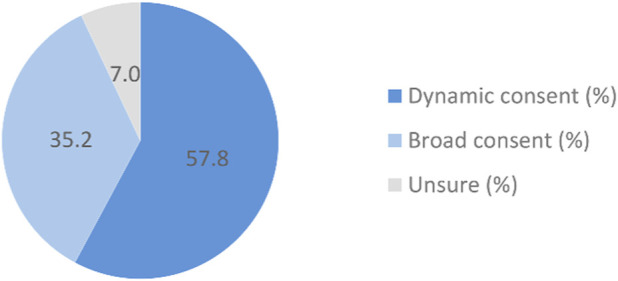
Preferences for consent models in the NPBBD (*n* = 1,027). The pie chart shows the consent models respondents preferred for the use of their personal biodata in biobank research.

With regard to consent model, as shown in Figure 14, 57.8% of the respondents favored a dynamic model, requiring renewed approval for each use of donated data in research, reflecting a preference for control and transparency. In contrast, 35.2% supported a broad consent model, in which a one-time approval would suffice for all subsequent studies. Thus, while some valued procedural efficiency, the majority preferred an ongoing, interactive consent process over a one-time formality.

## 4 Discussion

### 4.1 Summary of key findings

This study’s findings demonstrate that public perceptions of biodata use cannot be explained in simple dichotomous terms but involve a web of complex and interrelated factors. Respondents expressed significant concerns regarding the potential leakage of personal information while also maintaining high expectations for medical advancement. This tension between data protection and medical progress reflects the psychological dynamics of conditional trust. Individuals appear willing to share sensitive information if they perceive trustworthy protection systems. Trust can be secured not only through institutional frameworks but also through voluntary participation, understanding, and transparency.

Ambivalence was also evident in respondents’ motivations for participation. While many valued the public good of contributing to future generations and scientific progress, others emphasized personal benefits such as access to expert consultation and monetary compensation. This is noteworthy, as it suggests that public interest and personal benefit are perceived not as mutually exclusive or conflicting, but as coexisting and complementary values. Therefore, public engagement strategies could be more effective when framed to offer both social value and individual benefits. Given that most respondents preferred conditional participation (i.e., contingent on personal benefits) over unconditional public participation, it can be inferred that reward and return systems that extend beyond awareness campaigns could promote inclusive and sustainable engagement (Kim, 2023).

Recent developments in social sciences highlight how resource allocation significantly affects public acceptability and efficacy of policy initiatives. In line with this, respondents showed greater preference for substantive health-related benefits, particularly expert consultations, over one-time incentives such as coupons. This preference offers practical guidance for resource allocation, suggesting that future outreach should incorporate differentiated communication and incentive models aligned with participant priorities.

Respondents also prioritized contributions to scientific research and future generations over direct benefits for themselves or their families. However, this should not be interpreted as unconditional altruism but as a complex interplay of public benefit and personal gain, contingent on institutional trust. Willingness to participate for the public good can be realized only when supported by reliable governance and guaranteed data protection, underscoring the ambivalent structure of public attitude toward biodata use. This pattern of conditional trust is reflected in respondent views on governance as well. Most respondents favored a public-led model, with government and public institutions playing central roles and private medical institutions being allowed limited participation. These preferences highlight the importance of transparency, accountability, and public anchoring of governance rooted in public interest to ensure successful biobank operation. The respondents’ mistrust on private institutions may be stemming from concerns about profit-driven motives and commercial exploitation.

With regard to benefit-sharing, most respondents preferred that profits from biodata be reinvested in medical technology and data-driven research, rather than released for public welfare distribution. Moderate support was also expressed for policies that provide treatment cost assistance for rare and incurable diseases, indicating public openness to complementary policies that extent targeted support to disadvantaged groups. Business models should therefore incorporate transparent profit-flow structures that prioritize reinvestment while ensuring broader social contribution.

Finally, respondents displayed a selective attitude toward data provision, shaped by the perceived sensitivity of the information. Concerns about privacy and stigmatization were particularly pronounced with regard to lifestyle and behavioral data. Future project designs should provide more nuanced consent options by data type, with clear statements of purpose and strong anonymity safeguards. Moreover, withdrawal of consent should not be limited to full revocation but allow partial or time-bound withdrawal tailored to specific data elements. Consent process should be viewed not as a simple matter of choice, but as an issue of trust and the right to know. The participants’ preference for dynamic consent over broad consent appears to stem less from rejection of broad consent itself and more from misunderstandings, insufficient information, and concerns about autonomy. Therefore, broad consent could be made more publicly acceptable by coupling it with institutional trust and transparent information provision.

### 4.2 Practical implications

The UK Biobank, which is a leading case of successful broad consent implementation, has sustained trust through continuous and structured communication with participants (UK Biobank, 2022). Drawing from this case, the NPBBD can run a successful broad consent model by incorporating the key features of dynamic consent into a participant-centered, transparent data utilization structure. In special situations, such as significant changes in data use, highly sensitive data, or commercial applications, selective re-consent requests or notification-based feedback systems could be used as effective alternatives. Such an interactive, participant-controlled consent system could help improve public engagement than a single-consent approach.

This study also incorporated additional survey items not presented in the results using everyday scenarios—such as parcel delivery services—to explore contextual variation in perceptions of data leakage. Interestingly, respondents displayed relative indifference toward sharing personal data with general logistics services such as Coupang, although they were more cautious about providing consent for medical or research-related use of their biodata. This demonstrates that when complex factors such as purpose, controllability, and irreversibility of data use are at play, such as in the case of biodata use, privacy concerns become more salient. Such contextual sensitivity underlies ongoing public concerns around repeated consent in biobank participation. Therefore, while simple, one-time consent may be acceptable for clearly bounded contexts like parcel delivery, biodata use requires ongoing and iterative consent owing to uncertainty with regard to future applications.

Thus, the issue of consent extends beyond information provision to questions of how, to whom, and under what conditions information is shared, as well as the degree of participant control. Current challenges around biobank consent relate not only to procedures but also to the absence of an environment that fosters complete comprehension and trust, which is an institutional and communicative challenge. Securing public trust in biodata use therefore requires moving beyond consent acquisition, toward a substantive model of public engagement that treats the donors as equal partners in governance. To address concerns about misuse or commercial exploitation, biobanks must be presented not merely as scientific infrastructure but as platforms that strive to protect both individual rights and collective social values.

This complexity underscores the need for an integrated ELSI approach for genome-based public biodata projects. National initiatives involving highly sensitive information, such as genomic data, must be grounded on a multi-layered framework that includes scientific and medical outcomes, fair utilization of data, protection of participant rights, and cultivation of social trust. This orientation is already reflected in the NPBBD’s ELSI research objectives, which go beyond procedural consent to include structures such as establishment of ELSI ethics committees and advisory boards, citizen-participatory forums and public hearings, and monitoring of shifts in ELSI issue awareness (Lee, 2022). These measures show an institutional commitment to embedding public feedback into policymaking, positioning ELSI as a core mechanism for securing legitimacy and social acceptance. In formalizing the project, systematic internalization of this ELSI foundation will be essential.

International studies across the United States, Japan, and Korea have repeatedly highlighted that while most people recognize the research benefits of biobanks, actual participation tends to depend on reassurance about data protection, transparency, and the handling of genetic information (Yoshizawa et al., 2014). In the United States, respondents were generally divided over consent practices and showed concern about data misuse and the need for clear information (Sanderson et al., 2017). Meanwhile, studies in Japan reported cautious public engagement with a strong emphasis placed on secure consent models and trust in data management (Oikawa et al., 2023). Finally, findings from the present Korean survey similarly indicate that active participation requires robust privacy safeguards, transparent processes, and the fair sharing of research benefits. In all these countries, enhancing participant trust and clarifying consent procedures thus remain decisive for successful biobank engagement.

Ultimately, public engagement must serve as a structural foundation supporting both legitimacy and acceptance of biodata initiatives (Grežo and Sedlár, 2023; Schmanski et al., 2012). Effective engagement requires more than offering participation opportunities: it demands a policy feedback system that meaningfully solicits public voices, incorporates them into decision-making, and demonstrates visible influence on project direction. The ambivalent attitudes revealed in this study offer a critical insight: the public regards transparent, accountable, and public-led governance as a core prerequisite for biodata projects. This provides significant implications for designing policies aimed at strengthening participation in biobanks.

### 4.3 Conditional trust and data governance

The success of large-scale national bio-big data initiatives such as the NPBBD fundamentally depends on establishing conditional trust with the public, whereby participants’ willingness to share their information is predicated on perceived fairness, transparency, and accountability of the biobank’s data governance framework. Unlike absolute trust, conditional trust requires that institutions provide ongoing guarantees regarding data use, benefit-sharing, and the protection of participants’ rights. In this context, data governance must evolve beyond regulatory compliance to become a “trust-responsive” model, adapting procedural and substantive safeguards to address public expectations and ethical concerns.

Comparative analysis highlights how different countries institutionalize conditional trust through their governance models. In the United Kingdom, the Genomics England 100K Genome Project builds trust by incorporating transparency mechanisms such as citizen panels, multi-tiered data access, and robust feedback systems (O'Doherty et al., 2021). Meanwhile, Finland’s Findata system strengthens legitimacy through legal mandates and dynamic consent processes, ensuring that participants retain ongoing control over their data to guard against potential misuse (Findata, 2025). Japan’s BioBank utilizes hospital-centered ethical reviews and standardized data linkages to foster collective trust (Oikawa et al., 2023). Finally, in Korea, NPBBD’s multi-ministerial approach is promising but requires further reinforcement in aspects such as dynamic consent and transparent benefit-sharing to establish and maintain public confidence.

To institutionalize conditional trust, Korea must move toward multi-layered accountability structures, integrating legislative safeguards through participatory governance and technological protections Kim and Hong (2025). Dynamic consent, independent oversight, and fair benefit-sharing should be core design principles in NPBBD’s data governance. By prioritizing reciprocal benefits and procedural fairness, this initiative can transform public hesitancy into participatory confidence, laying robust ethical and social foundations for more precise medicine in Korea.

### 4.4 Limitations and future directions

Although this study offers valuable insights into public opinion, it has some limitations. First, although the sample was large, it may not fully represent the Korean population, limiting the generalizability of the findings. Second, unlike qualitative methods such as in-depth interviews, the survey design restricted exploration of respondents’ underlying reasoning behind their choices. Responses to hypothetical scenarios may not be accurately indicative of their natural behaviors in real-life contexts. Finally, exclusive reliance on web and mobile platforms may have introduced selection bias, characterized by an underrepresentation of older adults and individuals with limited digital literacy or internet access.

## Data Availability

The datasets presented in this study can be found in online repositories. The names of the repository/repositories and accession number(s) can be found below: https://drive.google.com/drive/u/0/folders/1A10DlVyKc-ce94IYR7eVlsH41gsPma_g.
