# Supplementary material for: Conditional trust as a driver of public engagement in Korea’s national project of bio-big data
Source: Front Genet. 2026 Jan 5;16:1713598. doi: 10.3389/fgene.2025.1713598 (PMC12812399; doi:10.3389/fgene.2025.1713598)
Supplement: Supplementary file 1 [file Table1.docx]

# Supplementary Table 1

**Table 3.** Respondents’ willingness to participate in the NPBBD classified according to demographic characteristics and perceptual factors (n=1,027).

| **Variable** | **Categories** | **Total** | **Willing: public good** | **Willing: personal benefits** | **Unwilling** | **p-value** |
| --- | --- | --- | --- | --- | --- | --- |
|  |  | **N** | **N (%)** | **N (%)** | **N (%)** |  |
| All |  | 1027 | 307 | 625 | 95 |  |
| Sex | Male  Female | 521  506 | 178 (34.2)  129 (25.5) | 310 (59.5)  315 (62.3) | 33 (6.3)  62 (12.3) | <0.001 |
| Age | 20-29 30-39 40-49 50-59  60 and above | 193 199  248  259  128 | 36 (18.7)  58 (29.1)  74 (29.8)  85 (32.8)  54 (42.2) | 144 (74.6)  128 (64.3)  151 (60.9)  146 (56.4)  56 (43.8) | 13 (6.7)  13 (6.5)  23 (9.3)  28 (10.8)  18 (14.1) | <0.001 |
| Awareness of the NPBBD | I know it  I have heard of it I don’t know it/I have not heard of it | 155 360  512 | 82 (52.9)  124 (34.4)  101 (19.7) | 70 (45.2)  213 (59.2)  342 (66.8) | 3 (1.9)  23 (6.4)  69 (13.5) | <0.001 |
| Participation in pilot project for Human Biospecimen Collection | Yes, have participated  No, have not participated  Unsure | 116  804  107 | 68 (58.6)  217 (27.0)  22 (20.6) | 48 (41.4)  504 (62.7)  73 (68.2) | 0 (0)  83 (10.3)  12 (11.2) | <0.001 |
| Awareness of the biobank | I have heard of it  I don’t know it/I have not heard of it | 504  523 | 201 (39.9)  106 (20.3) | 280 (55.6)  345 (66.0) | 23 (4.6)  72 (13.8) | <0.001 |
| Perceptions towards participation in the NPBBD | More concerns than expectations  Concerns and expectations are similar  More expectations than concerns | 159  461  407 | 24 (15.1)  102 (22.1)  181 (44.5) | 91 (57.2)  320 (69.4)  214 (52.6) | 44 (27.7)  39 (8.5)  12 (2.9) | <0.001 |
| Current enrollment in private health insurance | Enrolled Not enrolled Unsure/Do not remember | 836  120  71 | 268 (32.1)  29 (24.2)  10 (14.1) | 512 (61.2)  68 (56.7)  45 (63.4) | 56 (6.7)  23 (19.2)  16 (22.5) | <0.001 |
| Self-perceived health status | Poor  Fair  Good | 161  562  304 | 44 (27.3)  155 (27.6)  108 (35.5) | 103 (64.0)  358 (63.7)  164 (53.9) | 14 (8.7)  49 (8.7)  32 (10.5) | 0.068 |
| Experience of rare disease diagnosis (self or family) | None  Recovered after treatment  Currently undergoing treatment  Diagnosed but not receiving treatment | 892  42  52  41 | 255 (28.6)  18 (42.9)  18 (34.6)  16 (39.0) | 551 (61.8)  22 (52.4)  31 (59.6)  21 (51.2) | 86 (9.6)  2 (4.8)  3 (5.8)  4 (9.8) | 0.284 |
| Experience of rare disease diagnosis (self or family) 2 | Yes  No | 135  892 | 52 (38.5)  255 (28.6) | 74 (54.8)  551 (61.8) | 9 (6.7)  86 (9.6) | 0.052 |
| Experience of cancer diagnosis (self or family) | No history of diagnosis  Diagnosed and fully recovered  Currently under treatment  Diagnosed but not receiving treatment | 736  158  96  37 | 207 (28.1)  52 (32.9)  37 (38.5)  11 (29.7) | 455 (61.8)  97 (61.4)  52 (54.2)  21 (56.8) | 74 (10.1)  9 (5.7)  7 (7.3)  5 (13.5) | 0.212 |
| Experience of cancer diagnosis (self or family) 2 | Yes  No | 291  736 | 100 (34.4)  207 (28.1) | 170 (58.4)  455 (61.8) | 21 (7.2)  74 (10.1) | 0.085 |
| Number of household members | 1  2  3  4  5 or more | 133  187  353  301  53 | 34 (25.6)  50 (26.7)  113 (32.0)  91 (30.2)  19 (35.8) | 80 (60.2)  120 (64.2)  210 (59.5)  184 (61.1)  31 (58.5) | 19 (14.3)  17 (9.1)  30 (8.5)  26 (8.6)  3 (5.7) | 0.447 |
| Single-person household | Yes  No | 133  894 | 34 (25.6)  273 (30.5) | 80 (60.2)  545 (61.0) | 19 (14.3)  76 (8.5) | 0.076 |
| Marital status | Single  Married (including common-law)  Separated/Divorced/Widowed | 413  573  41 | 87 (21.1)  204 (35.6)  16 (39.0) | 284 (68.8)  320 (55.8)  21 (51.2) | 42 (10.2)  49 (8.6)  4 (9.8) | <0.001 |
| Experience of graduate school | Yes  No | 883  144 | 270 (30.6)  37 (25.7) | 537 (60.8)  88 (61.1) | 76 (8.6)  19 (13.2) | 0.149 |
| Employment status | Employed  Unemployed | 751  276 | 252 (33.6)  55 (19.9) | 442 (58.9)  183 (66.3) | 57 (7.6)  38 (13.8) | <0.001 |
| Perceived living standard | High  Medium  Low | 159  525  343 | 60 (37.7)  161 (30.7)  86 (25.1) | 93 (58.5)  319 (60.8)  213 (62.1) | 6 (3.8)  45 (8.6)  44 (12.8) | 0.002 |
| Monthly household income (KRW) | <2 million  2-4 million  4-6 million  6-8 million  ≥8 million | 86  268  264  219  190 | 14 (16.3)  75 (28.0)  82 (31.1)  62 (28.3)  74 (38.9) | 54 (62.8)  162 (60.4)  162 (61.4)  138 (63.0)  109 (57.4) | 18 (20.9)  31 (11.6)  20 (7.6)  19 (8.7)  7 (3.7) | <0.001 |
